# Reply to Rameix-Welti, “No Incongruity in Respiratory Syncytial Virus M2-1 Protein Remaining Bound to Viral mRNAs during Their Entire Life Time”

**DOI:** 10.1128/mBio.00629-19

**Published:** 2019-05-07

**Authors:** Thomas A. Edwards, John N. Barr

**Affiliations:** a School of Molecular and Cellular Biology, Faculty of Biological Sciences, University of Leeds, Leeds, United Kingdom; b Astbury Centre for Structural Molecular Biology, University of Leeds, Leeds, United Kingdom; Weill Cornell Medicine; Catholic University of America

## REPLY

First, we thank Marie-Anne Rameix-Welti for the thoughtful comments on our recent paper (1). The M2-1 protein from respiratory syncytial virus (RSV) has for many years been rather enigmatic in allowing researchers to probe its structure and function, with details slow to emerge. In Selvaraj et al. (2), we suggested a model that posited that the requirement of M2-1 in RSV transcription involved binding of an M2-1 tetramer to every viral mRNA transcript via its poly(A) tail, and one option we explored was that this interaction was maintained for the lifetime of the mRNA. This extends the conclusions presented by Rincheval et al. (3). However, we stated that a limitation of this model was that the abundance of M2-1 protein in the cell may be insufficient, governed by the relatively low abundance of M2 mRNAs that encode it. In the helpful letter (1) by Rameix-Welti about our paper (2), calculations are presented to suggest that molar quantities of M2-1 potentially exceed those of total viral mRNAs in the infected cell such that no incongruence exists. We appreciate these efforts to quantify the components of the proposed model which, although hypothetical, do lend support to its feasibility. Of course, we look forward to the time when such quantities can be experimentally determined.

Further to this, we would like to take this opportunity to bring to the debate an additional problematic scenario associated with this model to stimulate further discussion. During primary transcription, when the only available transcriptase and source of M2-1 is the RdRp bound to the infecting vRNA, our model posits that one tetramer of M2-1 is required for the generation of each RSV mRNA. As the M2 gene is the ninth transcriptional unit to be encountered by a transcribing mRNA, provision of newly synthesized M2 mRNAs to provide a pool of M2-1 protein would require a total of nine M2-1 tetramers to be brought into the infected cell. A critical gap in the current model is in the identification of the source of these additional M2-1 molecules.

The obligate requirement of M2-1 for transcription as revealed by extensive minigenome analysis suggests that an initial round of “M2-1-free” transcription cannot occur, so options include the repurposing of the M2-1 that is thought to be associated with the matrix in the virion or alternatively, the presence of multiple RdRps per genome. No experimental evidence for either of these possibilities currently exists.

As described above, the model we proposed has some notable gaps, but we view it as a starting point and hope the gaps may soon be closed following careful experimentation.

## References

[1] Rameix-Welti M-A 2019 No incongruity in respiratory syncytial virus M2-1 protein remaining bound to viral mRNAs during their entire life time. mBio 10:e00187-19. doi:10.1128/mBio.00187-19. 31064823 PMC6509182

[2] Selvaraj M, Yegambaram K, Todd EJAA, Richard CA, Dods RL, Pangratiou GM, Trinh CH, Moul SL, Murphy JC, Mankouri J, Éléouët JF, Barr JN, Edwards TA 2018 The structure of the human respiratory syncytial virus M2-1 protein bound to the interaction domain of the phosphoprotein P defines the orientation of the complex. mBio 9:e01554-18. doi:10.1128/mBio.01554-18. 30425144 PMC6234862

[3] Rincheval V, Lelek M, Gault E, Bouillier C, Sitterlin D, Blouquit-Laye S, Galloux M, Zimmer C, Eleouet J-F, Rameix-Welti M-A 2017 Functional organization of cytoplasmic inclusion bodies in cells infected by respiratory syncytial virus. Nat Commun 8:563. doi:10.1038/s41467-017-00655-9. 28916773 PMC5601476

